# Neurobiological Bases of Social Networks

**DOI:** 10.3389/fpsyg.2021.626337

**Published:** 2021-04-30

**Authors:** Mengfei Han, Gaofang Jiang, Haoshuang Luo, Yongcong Shao

**Affiliations:** ^1^School of Psychology, Beijing Sport University, Beijing, China; ^2^College of Education, Cangzhou Normal University, Cangzhou, China; ^3^Key Laboratory of Mental Health, Institute of Psychology, Chinese Academy of Sciences, Beijing, China; ^4^Department of Psychology, University of Chinese Academy of Sciences, Beijing, China

**Keywords:** social network, social cognition, mentalizing network, multilayer brain-social networks, neural mechanism

## Abstract

A social network is a web that integrates multiple levels of interindividual social relationships and has direct associations with an individual’s health and well-being. Previous research has mainly focused on how brain and social network structures (structural properties) act on each other and on how the brain supports the spread of ideas and behaviors within social networks (functional properties). The structure of the social network is correlated with activity in the amygdala, which links decoding and interpreting social signals and social values. The structure also relies on the mentalizing network, which is central to an individual’s ability to infer the mental states of others. Network functional properties depend on multilayer brain-social networks, indicating that information transmission is supported by the default mode system, the valuation system, and the mentalizing system. From the perspective of neuroendocrinology, overwhelming evidence shows that variations in oxytocin, β-endorphin and dopamine receptor genes, including oxytocin receptor (*OXTR*), mu opioid receptor 1 (*OPRM1*) and dopamine receptor 2 (*DRD2*), predict an individual’s social network structure, whereas oxytocin also contributes to improved transmission of emotional and behavioral information from person to person. Overall, previous studies have comprehensively revealed the effects of the brain, endocrine system, and genes on social networks. Future studies are required to determine the effects of cognitive abilities, such as memory, on social networks, the characteristics and neural mechanism of social networks in mental illness and how social networks change over time through the use of longitudinal methods.

## Introduction

Dynamic and intricate personal relationships in daily life compose our social world. Adapting to the complexities of the social world is a critical component of people staying healthy and maintaining well-being (Schmälzle et al., 2017; Pearce et al., 2017). A social network refers to the structure of the social world that provides theoretical and methodological paths for researchers to comprehensively investigate diverse patterns of interconnections in the social world (Pillemer et al., 2017; Falk and Bassett, 2017). The in-depth exploration of the neurophysiological mechanism underlying social networks is crucial for understanding the preferences of individuals in constructing social networks and the reasons/mechanisms underlying the effect of social networks on promoting physical and mental health (Pillemer et al., 2017).

In contrast to traditional social psychology, which focuses on mutual relationships (e.g., friends, couples, and colleagues), social networks provide relatively complete and ecologically valid structural and functional frameworks that integrate multiple levels of interaction units, including one-to-one social relationships among individuals and their indirect relationships (Fischer, 1983; Krause et al., 2009; So et al., 2015). The social network supports interpersonal exchange and communication of emotions, information, and material (Blumen and Verghese, 2018; Bang et al., 2019). By providing structural information, social network indicators, including the network size, network complexity and core-periphery role in the network, further reflect the extent to which individuals blend into their social environments (Blumen and Verghese, 2018; Bang et al., 2019). For example, social network analysis provides a deeper understanding of social complexity by directly measuring the number or types of social relationships. Overwhelming evidence indicates that the complexity of the social network structure is positively correlated with social skills, such as sociability, mentalizing or encoding biological motion (Wey et al., 2008; Kirby et al., 2018; Gentina et al., 2020). Based on a complete map of the asocial network of interaction units, the social network can also describe the source and amount of social influence (e.g., social support, conformity, imitation and compliance) perceived by individuals (Blumen and Verghese, 2018; Bang et al., 2019). For example, social network analysis encourages researchers to consider the social background of an individual. The complexity of an individual’s social network structure can positively predict perceived social support, subjective happiness, immune function, cognitive function and exercise behavior, and negatively predict depression and anxiety and the risk of cardiovascular disease (Bryant et al., 2016; Joo et al., 2017a; Ali et al., 2018; Kim H. et al., 2019). In addition, the influence of an individual, such as loneliness (Cacioppo et al., 2009), depression (Bryant et al., 2016), ideas (Scholz et al., 2017), smoking habits (Rosenquist et al., 2011), obesity (Christakis and Fowler, 2007), and physical activity (Aral and Nicolaides, 2017), can also spread to direct and indirect friends throughout social networks. Therefore, social network analysis has contributed novel insights by condensing individuals’ social functions into structural indicators, making the measurements of individuals’ social functions simpler and more comprehensive. Recent advances in the neurobiological mechanisms of social networks complement advances in social neuroscience that have described how an individual’s brain, neuroendocrine system and genes interact with their personalities, behaviors and their ability to adapt to the social environment (Falk and Bassett, 2017).

Studies of network neuroscience started from the “social brain hypothesis” proposed by Brothers in 1980s (Brothers, 1990). Primates usually live in large and complex social groups and need a “larger” social brain to process complex social information for their adaptation to the social environment (Brothers, 1990; Dunbar, 2012). In earlier studies, many researchers investigated the relationship between the social brain and social environment in primates and confirmed that the social complexity of primates, including social group size, complexity of the male mating strategy, and allied strategies, is closely related to the cerebral cortex size (Sallet et al., 2011). In recent years, similar results were reported in human studies. A human individual’s social network structure and network information transmission are closely related to the size and functional connectivity of the social brain (Dunbar, 2012; Falk and Bassett, 2017; Kwak et al., 2018). Furthermore, a growing body of research underscores the important role of neuropeptides and polymorphisms of their receptor genes on an individual’s social network. For instance, the capacity to maintain complex social networks has been linked not only to oxytocin, β-endorphin and dopamine but also to gene variations in oxytocin receptors, β-endorphin receptors and the dopamine receptor 2 gene (Pearce et al., 2017, 2018). These recent advances have mapped multiple layers of neurophysiological mechanisms underlying social networks, which holds great promise to improve our knowledge of how an individual’s social network represents his/her social functions and how that social network is constructed and maintained.

Recent research on the neurophysiological mechanisms of social networks has mainly focused on the relationships among structural and functional properties of social networks in the context of the brain, the neuroendocrine system and genes. Structural properties describe the structure of the network, including the size and complexity of social networks (Bickart et al., 2011; Falk and Bassett, 2017). Functional properties are associated with information transmission within social networks, e.g., how an individual’s emotions, values, and behavior influence others throughout the social network (Falk and Bassett, 2017). Here, we review recent studies that focus on the working patterns of the brain, the endocrine system and genes, with insights from social network analysis. Starting from the structural and functional properties of social networks, we first elucidate the relationship between social networks and the social brain, including the connections of the network structure with brain structure and function, the regulatory role of the network structure in the social cognition process, and the multilayer brain-social networks associated with network functional attributes. Subsequently, we summarize the relationship between social networks and the neuroendocrine system and genes. Based on all the theories and methods of social network analysis, we propose that social networks provide a relatively comprehensive description of people’s social systems and social functions. By integrating studies of neurophysiology linked to social networks, we will obtain novel insights into how the brain, the endocrine system and genes shape social behaviors, and how social context influences group behaviors. In addition, we will obtain novel insights into the prediction, identification and intervention in diseases related to abnormal social function. Finally, based on recent studies, we speculate that an individual’s social network may require the functions of the combination of the social brain, the neuroendocrine system and genes. We summarize the related studies and provide basic and directional guidance for future research.

## Neural Bases of Social Network Structure

**FIGURE 1:**
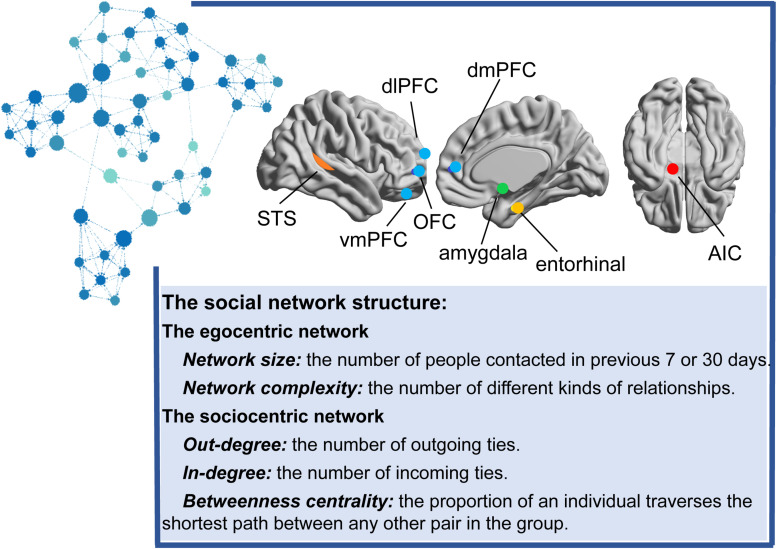
The main brain regions correlated with the social network structure. Different colored dots in the brain indicate different brain structures. Blue nodes indicate the mentalizing network, including the vmPFC (ventromedial prefrontal cortex), OFC (orbitofrontal cortex), dlPFC (dorsolateral prefrontal cortex) and dmPFC (dorsomedial prefrontal cortex); the orange dots indicate the mirror network (superior temporal sulcus, STS); the green dot indicates the amygdala; the yellow dot indicates the entorhinal cortex; and the red dot indicates the AIC (anterior insular cortex). Representative graph of the sociocentric network organized for approximately 52 college students. Each node represents one person. Lines between nodes denote relationships. The node color and size indicate the degree centrality (the sum of the in-degree and out-degree ties). Larger nodes in dark blue indicate more outgoing ties and incoming ties, and smaller nodes in light blue indicate fewer outgoing ties and incoming ties. According to the review, the social network size or complexity is positively correlated with the structure and activation of amygdala, the mentalizing network, the mirror network, entorhinal cortex and AIC. The limited research on the sociocentric network only emphasized the association between the degree/betweenness centrality and amygdala or the mentalizing network.

Previous studies have employed various metrics to describe the social network structure, such as the number of regular relationships that a person maintains over a 7-day or 1-month period, reflecting the network size (Bickart et al., 2011, 2012), or the number of different types of these relationships to which an individual belongs, reflecting network complexity (Cohen et al., 1997; Bickart et al., 2011, 2012). Network size and complexity are focused on the individual egocentric network, in which only direct social ties to the focal individual are involved. Recently, an increasing number of studies has started to focus on both direct and indirect social ties from a sociocentric perspective (Smith and Christakis, 2008). The sociocentric network is constructed from all relationships between every two members in a specified group, such as a class, club, or town. Specifically, the number of outgoing ties (out-degree centrality), incoming ties (in-degree centrality), and the proportion at which an individual frequently lies on the shortest path between any other pair in the group (betweenness centrality) are usually used to reflect the whole structure of the network (see Figure 1).

Another important feature of living in a social group is that individuals adapt and utilize their different social status/hierarchies to acquire relative social resources. Social status forms based on genetic or cultural background and plays a key role in determining the relationships among the group members (Dávid-Barrett and Dunbar, 2012). An individual’s social status can affect how people navigate their social world, including effects on their social cognitive processes or social contacts (Muscatell et al., 2012). In contrast to the social network indicators mentioned above, which mainly focus on the roles of social integration (e.g., the degree to which people are embedded in social relationships), social status emphasizes the role of people’s social power in controlling the flows of information and the access to social resources through social relationships (Hussong et al., 2019). However, social status and social network structure are often highly and positively correlated, and they are different dimensions of social position (Hussong et al., 2019; Rueden et al., 2019). For example, low-status individuals may have high-quality but fewer social relationships, whereas relatively high-status individuals usually engage in fewer close relationships but have more social relationships (Berkman et al., 2000; Kraus and Keltner, 2009). Thus, social status is important to consider as a part of the social network structure. However, social status is not a typical index of social network analysis. Because it differs from social network centrality, which is a more objective measure acquired from an individual’s actual social relationships, social status is more like an intrapersonal indicator that is usually measured with a subjective questionnaire, socioeconomic status (SES) (e.g., educational attainment and occupational status) or performance ranking in virtual competition scenarios in human studies and measured less frequently using peer nomination or peer ranking (Hu et al., 2016; Okamoto et al., 2017; Rueden et al., 2019). In the present study, we review the social network and social status studies to obtain a deeper understanding of social context.

Remaining social roles in the network depend on the capacity of social cognition, including the identification of social emotion, understanding of social cues, and theory of mind. Evidence from social neuroscience has suggested that the volume and activities of brain regions (e.g., amygdala and mentalizing network) involved in social cognition processes are also related to the social network structure.

### Amygdala

The amygdala, a core brain region involved in the emotional network, plays an extremely prominent role in processing and handling social information such as emotion-related social signals, social values, motivation, and identity (Adolphs, 2001; Gothard et al., 2018). The earliest study focusing on the relationship between the amygdala and social behavior used primates as study subjects and found that after amygdalectomy, the individuals who formerly held a high social status in the group had a decreasing social rank and became extremely compliant (Rosvold et al., 1954; Watanabe and Yamamoto, 2015). Accordingly, Munuera et al. (2018) further reported that the primate amygdala encodes the hierarchical rank and reward value. Similar to primate research, a growing body of literature on humans has also shown that the amygdala is associated with rank in one’s social network. Bickart et al. (2011) examined the total number of regular contacts that a person maintains (social network size) and the number of embedded networks (social network complexity). They reported that these two social network indexes positively predicted the amygdala volume. Likewise, Kanai et al. (2012) tested a larger sample and found that the gray matter density in the amygdala correlated with both online and real-world network sizes, whereas the gray matter density in the right superior temporal sulcus (STS), left middle temporal gyrus and entorhinal cortex was specifically associated with online social network size. In another study using three social network indicators to further confirm the relationship between the amygdala and social network structure, researchers found that the online network size (the number of Facebook friends), offline network size (the number of regular contacts of an individual in the last 30 days), and social support network size (the number of friends who could provide social support) were positively correlated with the gray matter density of the amygdala and the right entorhinal/ventral anterior temporal cortex, regardless of whether the individuals were in a resting state or viewing the faces of friends or strangers (Heide et al., 2014). The important role of the amygdala in social network construction and maintenance has also been confirmed by Jones et al. (2020). According to the authors, the amygdala is presumed to track visual signals in social interactions, such as face stimuli, gestures, and expressions (Bickart et al., 2011, 2012). A larger amygdala provides an individual with advantages in processing non-verbal social signals (Bickart et al., 2011, 2012). An amygdala with a larger volume and higher gray matter density enables an individual to search, decode, and match multilayered and complex social signals for processing more complex social network information (Bickart et al., 2011, 2012). In addition, the amygdala tracks the reward value brought by social interaction. Individuals with a larger volume, higher gray matter density, or higher activation level of the amygdala tend to perceive social interaction as more interesting and of higher reward value, which in turn prompts them to develop more social connections (Bickart et al., 2012; Zerubavel et al., 2015; Liu et al., 2019).

Brain structure and function are not independent of each other. Instead, structural changes in the cerebral cortex usually cause corresponding functional changes (Falk and Bassett, 2017). Therefore, a number of studies searched for more evidence at the level of brain functional connectivity to determine the importance of amygdala networks in social network construction. The functional connectivity between the amygdala and orbitofrontal cortex (OFC) is crucial for facial expression recognition, social strategy development, social reward processing, prosocial behavior, etc. (Hampton et al., 2016; Kwak et al., 2018). Researchers generally agree that amygdala-OFC functional connectivity stably and positively predicts the differences in social network size among individuals (Hampton et al., 2016; Kwak et al., 2018). According to a previous study, smell is a type of social signal that conveys information about an individual, such as sex, disease and emotional state; therefore, individuals with high olfactory sensitivity are able to identify social signals from the body odor of others, which is conductive to social interaction (Zou et al., 2016). Individuals with higher functional connectivity between the amygdala and OFC, which are the brain regions related to olfactory sensitivity and theory of mind, are more sensitive to olfactory signals and have a larger social network size (Zou et al., 2016). Furthermore, Bickart et al. (2014) divided the amygdala into three subareas and investigated the relationships between the social network size and the intrinsic anatomical connectivity levels of the three subareas with other brain regions. The functional connectivity levels of the perception network, which consists of the ventrolateral amygdala and OFC and is responsible for decoding social signals, and the social affiliation network, which consists of the medial amygdala and ventromedial prefrontal cortex (vmPFC) and is responsible for processing social reward information, are positively correlated with social network size. In addition, the functional connectivity level of the aversion network, which is composed of the dorsal amygdala, insula and hypothalamus and is responsible for processing aversive stimuli, is not significantly correlated with social network size (Bickart et al., 2012, 2014). This finding suggests that the amygdala does not work independently. Instead, it works synergistically with other brain regions to enhance an individual’s ability to process social information. However, the perception network, the social affiliation network and the social aversion network were defined by a data-driven approach, and the intrinsic functional connectivities among them have not been verified in further studies (Bickart et al., 2012).

A relatively stable correlation has been identified between social networks and the gray matter structure and activation level of the amygdala, a region of the brain that has attracted attention from researchers for many years. The aforementioned studies support the hypothesis that the volume and activation level of the amygdala may affect the social function and social network structure of human individuals by affecting their abilities to process social signals and perceive social reward value. The amygdala comprises multiple nuclei that are distinct in morphology, histochemistry, cytoarchitecture, and functional connectivity (Kedo et al., 2018; Jones et al., 2020). Jones et al. (2020) further found that the central nucleus, basal and accessory basal nuclei of the basolateral amygdala are significantly associated with social network size, but no association with the lateral amygdala nucleus was found. However, the participants in this study were homeless and precariously housed people, not the general population. In addition, the social network indicators of this study are based on a sociocentric network, which is different from the studies described above that focused on an individual’s egocentric network. The egocentric network focuses on the number and types of all direct social relationships associated with the core individuals, while the sociocentric network focuses on the social position of individuals in a specific collective based on both their direct and indirect relationships. The egocentric network and sociocentric network describe the network structure from micro level and macro level, respectively. Therefore, these two different network indicators may reflect different brain areas and brain activities. Future studies should explore the sociocentric network to supplement the neural basis of social network at the macro level.

Indeed, some studies failed to replicate the important role of the amygdala in a social network (Lewis et al., 2011; Powell et al., 2012; Noonan et al., 2018; Spagna et al., 2018). According to the authors, social networking is complicated and depends more on social cognition abilities, which may be related to frontal and temporal structures, rather than to non-verbal social signal identification. These studies did not obtain consistent results for the amygdala, possibly because they assessed the social network index using distinctly different methods. Thus, the relationship between the amygdala and the social network must be verified in further studies using consistent measurements and populations. In addition, the studies described above discussed only resting-state networks, and therefore, more studies are needed to clarify the causal relationship among the amygdala, social function, and social network structure in the future.

### The Mentalizing Network

The amygdala network is involved in the perception and understanding of non-verbal social signals. However, the social cognitive ability required for establishing and maintaining social networks is beyond the superficial processing of social signals; instead, the deep processing of the psychological state of other people is also key to successful social interactions (Kanai et al., 2012). Premack and Woodruff (1978) proposed the concept of “theory of mind” to describe an individual’s social cognitive ability, which refers to an individual’s ability to infer the personality characteristics, psychological state, and intentions of other people. Studies have found that the core brain regions involved in theory of mind include the medial prefrontal cortex (mPFC), the temporal-parietal junction (TPJ), the posterior cingulate cortex (PCC), the medial OFC (mOFC), and the precuneus, which are commonly known as the “mentalizing network” (Mitchell, 2009; Muscatell et al., 2012). According to these findings, the network structure of an individual is postulated to be limited by mentalizing competence. In recent years, researchers have also begun to explore evidence supporting the hypothesis that theory of mind reflects the structure of an individual’s social networks from the perspective of the mentalizing network.

Among the core brain regions in the mentalizing network, the covariation in the PFC and social network structure have been emphasized in most studies. The PFC is the core brain region in the mentalizing network. The vmPFC and OFC are involved in the emotional part of the theory of mind and are mainly responsible for understanding the emotional state of others (Abu-Akel and Shamay-Tsoory, 2011). The dorsomedial PFC (dmPFC) and the dorsolateral PFC (dlPFC) participate in the cognitive part of the theory of mind and are mainly responsible for inferring the beliefs and intentions of others (Abu-Akel and Shamay-Tsoory, 2011). Existing studies have revealed that both emotional and cognitive components are reflected by the social networks of individuals. Lewis et al. (2011) have found that individuals with a larger social network size in the previous 30 days understand the psychological state of others more accurately and generally have larger gray matter volumes in the mOFC and vmPFC (Kanai et al., 2012). In another study, researchers further explored the possible causal relationship among social network size, theory of mind, and the PFC in human individuals using a path analysis. They found that theory of mind plays an intermediary role between the OFC volume and social network size. Individuals with larger OFC volumes have higher mentalizing competence and thus more complex social relations (Powell et al., 2012). Kwak et al. (2018) constructed sociocentric networks with town as the unit and found that the volumes of the dmPFC, OFC, and TPJ were generally larger in the individuals with higher in-degrees. Despite the discrepancy in results, all the aforementioned studies emphasize the significant role of the OFC. Compared with the cognitive component of social cognition, the emotional component may be more important for social network construction and maintenance in humans. However, this hypothesis should be confirmed by performing additional research.

Researchers have explored the relationship between mentalizing network functional activity and social networks. A study conducted by Pillemer et al. (2017) included elderly populations. The elderly participants were asked to report the number of social ties with high contact frequency (network quality) and the number of social ties that the participant recently contacted (network quantity). The researchers found that the functional connectivity level between the frontal and parietal lobes, mainly including the dmPFC, dlPFC, PCC, and precuneus, was positively correlated with the two social network indicators listed above. Network quality was positively correlated with the functional connectivity level of the lateral part of the frontoparietal network, and network quantity was positively correlated with the functional connectivity level of the medial part of frontoparietal network (Pillemer et al., 2017). Noonan et al. (2018) also found that the functional connectivity levels between dmPFC and dlPFC with the anterior cingulate cortex (ACC) predict the social network size of an individual (Noonan et al., 2018). In another study on elderly individuals, those with greater functional connectivity between the frontal and temporal lobes were shown to have larger social networks. Frontotemporal functional connectivity is closely related to an individual’s ability to perceive the agreeableness of surrounding people, suggesting that individuals with larger social networks may perceive greater interpersonal closeness (Bang et al., 2019). Thus, although some brain regions play important roles in social behavior, extensive brain functional connectivity is likely more important for social network construction and maintenance.

Theory of mind is the basic mechanism through which individuals successfully establish social relationships. By examining the relationship between social networks and the core brain regions of the mentalizing network, researchers have shown that social networks and theory of mind share the same neural circuits. However, Bickart et al. (2012) found that the functional connectivity of the mentalizing network, including the dmPFC, precuneus and TPJ, was not related to either the social network size or complexity, and the authors speculated that the contribution of the affective processing by the amygdala to social networking was greater than social cognition (Bickart et al., 2011, 2012). Nevertheless, they could not ignore the important role of the mentalizing network. The authors emphasized that the functional connectivity between the amygdala and the vmPFC and OFC predicted social network structure.

### The Mirror Neuron System

The mirror neuron system, another important neural system involved in social cognition, is also related to social network structure. The mirror neuron system is mainly responsible for supporting imitation and understanding other people’s actions (Ikeda et al., 2019). Brain regions such as the inferior frontal gyrus (IFG), inferior parietal lobule (IPL), and STS are involved in the mirror neuron system. However, the identification of biological motion supported by the mirror neuron system is a basic and vital function for individuals’ sociality, and only a few network neurosciences studies have focused on the mirror neuron system. They all emphasized the association between social network properties and the posterior STS (pSTS). The pSTS is specialized for understanding and imitating the non-verbal social signals of others, such as body movements, eye gaze, and mouth movements (Deen and Saxe, 2019). Researchers reported a positive correlation between the online network size (number of friends on Facebook) with the pSTS gray matter density, and real-world social network size did not have a significant relationship with the pSTS (Kanai et al., 2012). Other studies were based on task-state functional magnetic resonance imaging (fMRI) when identifying biological motion. Dziura and Thompson (2014) investigated the effects of the social network size and complexity on the perception of basic, non-verbal social stimuli (e.g., gestures and expressions) and found that the activation of the STS and amygdala induced by biological motion recognition was positively correlated with social network indicators. Based on this result, the individual at the center of a network must have a strong ability to identify obscure social signals in order to play a complex social role (Dziura and Thompson, 2014). Accordingly, Kirby et al. (2018) investigated neural sensitivity to biological motion related to social network properties in middle childhood. Consistent with the study by Dziura, the children’s social network size (the number of people the child regularly sees or talks to), diversity (the number of social roles the child plays) and embeddedness (the number of social domains in which the child is active) predicted activation in the pSTS, which significantly correlated with the neural sensitivity to biological motion when the children viewed biological motion.

Surprisingly, few social network neuroscience studies emphasize the function of the mirror neuron system in social networking. However, the existing research has provided evidence that the ability to construct and maintain social networks is limited by neural sensitivity to biological motion. Little direct evidence is available on how brain structure and functional connectivity among regions in the mirror neuron system influence social networking. This influence is important because the ability to understand biological motion, including facial expressions and body movements, can affect an individual’s social skills and the quality of social interaction (Oberman et al., 2007). Recognizing and clarifying the relationship between the mirror neuron system and the social network (not only social cognition) might improve our ability to predict group behaviors (Freiwald, 2020). Thus, research in this area is needed for further and deeper explorations of the mirror neuron system and social networks in the future.

### Other Brain Regions

In addition to the involvement of the amygdala, the mentalizing network and the mirror neuron system in social network construction and maintenance, previous studies have found that the entorhinal cortex and anterior insular cortex (AIC) are also closely related to the social network structure in humans. By comparing two different samples from New York and Beijing, researchers found that social network size and complexity positively correlate with AIC volume (Pillemer et al., 2017; Spagna et al., 2018). One of the important roles of the AIC is to process interpersonal emotional information, including sympathy, empathy and understanding the feelings of others (Pillemer et al., 2017; Spagna et al., 2018). Previous studies also revealed a linear correlation between the online social network size and entorhinal cortex gray matter density (Kanai et al., 2012; Heide et al., 2014). The entorhinal cortex is related to memorizing names and faces, but unrelated to social cognition (Sperling et al., 2001; Kanai et al., 2012). Thus, memory ability is also clearly one of the indispensable abilities for individuals to maintain a large social network. In other words, social cognitive ability is not the sole factor limiting the social network structure of an individual. Accordingly, Joo et al. (2017b) examined the global structure of the sociocentric social network in an entire village and suggested that older adults’ social network embeddedness (number of social groups to which an individual belongs), but not social size, was positively associated with functional connectivity between anterior-posterior regions, including the right inferior frontal gyrus and the occipital lobe (right lateral occipital cortex), the cingulate gyri and parietal (left superior parietal lobule and precuneus cortex) and occipital lobes (right lateral occipital cortex). Older adults with higher network embeddedness may need more cognitive resources to deal with complicated social relationships, which may subsequently activate functional connectivity among anterior-posterior regions.

In summary, social network construction and maintenance involve various related brain regions, including the amygdala network, the mentalizing network, the mirror-neuron system, the entorhinal cortex, and the AIC (see Figure 1). Although the aforementioned studies used different social network indicators and measured the indicators for different periods of time, ranging from 1 week to 1 month, and from an individual egocentric network to a sociocentric network, they reported relatively reproducible findings. However, Lin et al. (2019) repeated previous studies and did not identify a brain structure that was significantly related to social network structure. The authors postulated that the relationship between dynamic indicators of social network structure and brain function, instead of brain structure, should be emphasized because social networks are dynamic, as social relations might naturally increase or decrease over time, but the brain structure is relatively stable (Lin et al., 2019). Although many of the studies described above have discussed the functions of brain regions, they only focused on resting-state networks, and few of them mention how social networks affect the processing of social information by the brain. More in-depth studies should be conducted to investigate task-state networks and to explore and verify the regulatory effect of network structures on neural function.

### Brain Connectivity Dynamics During Social Information Processing Reflect the Social Network Structure

The position of an individual in a social network represents the individual’s social resources, preferences, and status. Individuals with larger or more complex networks have more opportunities to influence others or be affected by others, which provides those individuals with abundant social experience that guides them to cope more effectively with problems in different social situations (O’Donnell et al., 2017). Therefore, social network structure may further affect the activation level of the related brain regions during social information processing. In recent years, researchers have used various social cognitive tasks to explore how social networks regulate brain activity in the process of social cognition.

In a study investigating social conformity, O’Donnell et al. (2017) presented a series of software applications to study subjects and asked them to what extent they would recommend the apps to their friends. Additionally, the authors investigated the whole network formed by each individual and his/her friends on Facebook. Compared with the individuals with lower betweenness centrality (individuals with fewer opportunities to pass information and those on the outer edges of the social network), the individuals with higher betweenness centrality exhibited greater activation of the mentalizing network when they disagreed with their peers. Thus, when providing suggestions to others, the individuals in the center of the network care more about other people’s opinions; i.e., they are more sensitive to the opinions of others (O’Donnell et al., 2017).

Researchers asked participants to complete a classic Cyberball social exclusion task and used social network density as a network indicator to evaluate interpersonal communication. Friends of individuals with a higher network density are usually friends with each other. Compared with individuals with a higher network density, the functional connectivity between the left and right TPJ was higher in individuals with a lower network density when they were rejected. The researchers postulated that the individuals with a lower network density thought more about the causes and consequences of rejection and conducted more self-reflection when they were rejected by others, while those with a higher network density were less affected by social exclusion because their close social connections helped them buffer the negative effect of social exclusion (Schmälzle et al., 2017). Based on this observation, social networks affect interpersonal interaction patterns. Nevertheless, social networks and interpersonal interaction patterns might have a mutual relationship, which requires further investigation.

Previous studies emphasized the important role of social experience in processing social reward value. As shown in the study by Meshi et al. (2013), the activation of the ventral striatum when participants gain reputation from others positively predicted the degree of Facebook use (e.g., the number of friends on Facebook or the minutes per day spent on Facebook). Meanwhile, the activity in the ventral striatum during monetary gain did not predict Facebook use. The ventral striatum is a well-established structure involved in processing rewards such as food, money, and social stimuli (Meshi et al., 2013). The social experience/context created by our social network may influence our perception of social rewards based on positive evaluations from others (Fareri and Delgado, 2014).

In a recent study, researchers revealed that social network structure also moderates neural function in a non-social task (Go/No-Go response inhibition task). Adolescents with larger online social networks who are involved in more communities in their social network showed a stronger relationship between response inhibition and functional connectivity in self-referential systems (e.g., vmPFC) and response inhibition systems (e.g., superior temporal gyrus), suggesting that social context might facilitate self-control in adolescents. The real-life social network might have accounted for the compensatory role of social experience for better task performance (Tompson et al., 2020). However, adolescents are more sensitive to social influence than adults. Thus, the compensatory role of social experience in self-control does not extend to adults.

The abovementioned findings lay an important foundation for studies investigating how social network structure relates to neural sensitivity to social information processing. In addition, some studies found that the activation of an individual’s brain regions related to the processing of another person’s information is affected by the position of the other person in his/her social network. For example, using two small groups (13 members) as research subjects, a study based on whole networks found that the activation levels of the dmPFC, precuneus, and TPJ in the mentalizing network increased when the individuals saw the face of a highly popular member in their group, in which the reward system (the vmPFC, amygdala, and striatum) played an intermediary role. In other words, the individuals perceived that the popular member would bring them higher reward value, which in turn prompted them to infer the psychological state of the popular member (Zerubavel et al., 2015). In another study, participants were asked to watch short self-introduction videos of their friends, friends’ friends, and friends of friends’ friends in their whole network. Watching videos of their immediate friends, the participants had high activation levels in the lateral superior temporal cortex (STC), mPFC, and IPL, which are the brain regions generally responsible for processing physical distance and abstract distance (e.g., social distance) information from the individuals themselves. Thus, individuals will spontaneously process information related to the relationship between others and themselves in a network, regardless of whether the task of social distance judgment is required (Parkinson et al., 2017).

Studies that used different social cognitive tasks all confirmed that an individual’s social network structure affects his or her ability to process social information. The individuals at the core of a network have rich social experience and use the mentalizing network differently from those on the edge of the network in social cognitive tasks (Falk and Bassett, 2017). However, these studies only used social network indicators as moderating variables and did not manipulate them. Future research can use social networks as independent or dependent variables for in-depth investigations to further clarify the relationship between social networks and social cognition.

### Neural Bases of Social Status

High-status individuals typically have more opportunities to receive primary resources and are engaged in communicating with others for longer periods (Fiske, 1992; Chiao et al., 2009). Navigation of the social world with a higher hierarchical status requires higher level of capacities in expressing and recognizing social status signals, as well as processing cognitive information (Chiao et al., 2009; Hanson et al., 2013). MRI studies have found that a lower SES is associated with decreased gray matter or volumes in prefrontal, temporal, and parietal cortices, the hippocampus and amygdala (Hanson et al., 2013; Noble et al., 2013; Brito and Noble, 2014; Finn et al., 2016; see Willard and Shively, 2016 for review). Researchers have attempted to discover the neurocognitive function of social status using behavioral tasks, including social information processing, working memory, language processing, social information processing, etc. For instance, Muscatell et al. (2012) revealed that college students’ subjective social status in the university community was negatively correlated with neural activity in the dmPFC, mPFC, and precuneus/PCC (posterior cingulate cortex) when processing social information in response to negative feedback. Adolescents with a lower SES showed greater neural activity in the dmPFC and amygdala under social threats. Some other fMRI studies focused on SES revealed similar results. For instance, children and adolescents with a higher SES showed less overall activity in several areas associated with executive function, including the inferior frontal gray and the dorsal anterior cingulate cortex (dACC), when performing tasks related to working memory, mathematics achievement, language processing, etc. (Sheridan et al., 2012; Finn et al., 2016; see Farah, 2017 for review). Individuals with a low social status may require more attention on cognitive information processing, which would be reflected in greater activities in the brain regions discussed above, compared to people with a higher SES. In addition, fMRI studies also reported differences in reward-related brain regions (e.g., ventral striatum and caudate) between individuals with high and low SES, and the latter were more sensitive to reward associated learning and actions (Yaple and Yu, 2019). However, individuals with a low social status do not always display hyperactivity in executive network regions and reward-related regions (Finn et al., 2016; Yaple and Yu, 2019). In another fMRI study, social status was measured by adolescents’ popularity and acceptance in their class. Their popularity and acceptance were analyzed by asking adolescents which classmate they like most and least and which classmate they perceive as most and least popular. Adolescents who were nominated as most likable and popular were central and influential in a peer group with a high social status. Participants’ acceptance was positively correlated with activity in the dorsal anterior cingulate cortex (dACC) during exclusion, while participants’ popularity was positively associated with activity in the ventral striatum and mPFC during exclusion (Water et al., 2017). Therefore, when individuals with different social statuses process information, the social status will be reflected in the activities of different brain regions. The relationship between social status and the activities of these brain regions may be affected by the types of processed information and the measurements of social status. Numerous studies have focused on the neural mechanism of social status, but few directly compare the similar and distinct neural mechanisms between social status and social network. According to the results mentioned above, social status shares overlapping neural representations with social network structure, such as the mPFC and amygdala.

However, much of the work on social status and brain function has focused on SES. Although SES is an objective indicator measured by the income or education level of an individual/family, it does not involve the description of social relationships. SES is quite different from the index of social network structure, making a direct comparison of the neural basis of these two social structure-related indicators difficult. Social status has also been measured using peer ranking or nomination, depending on the social network (Water et al., 2017; Rueden et al., 2019). Future research should increase the exploration of the relationship between the neural mechanisms underlying social network structure and social status, such as whether the correlation between social status and brain activities will be moderated by social network indicators.

## Neural Bases of Social Network Function

Social network structure directly affects the physical/mental health and behavior of individuals. Some health-risk behaviors in social networks also affect the physical/mental health of individuals through interpersonal transmission, and the key members’ opinions toward health affects other members in a social group (Christakis and Fowler, 2007; Platt et al., 2014). Therefore, the topics regarding how information is transmitted in a social network and whether the form of transmission is restricted by neural networks have attracted interest from researchers.

**FIGURE 2:**
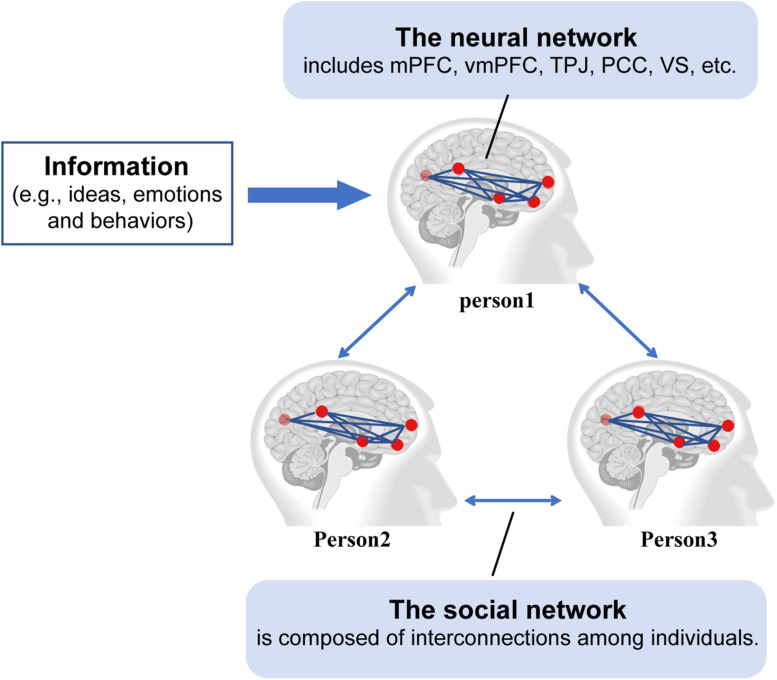
Multilayer brain-social networks. Information (e.g., ideas, emotions, and behaviors) processed in one brain region in person 1 can be transmitted to another region in person 1 via the neural network. The transmission of information can lead to a change of opinion in person 1, which can then be transmitted from person 1 to person 2 and person 3 via the social network. mPFC refers to the medial prefrontal cortex, vmPFC refers to the ventromedial prefrontal cortex, TPJ refers to the temporo-parietal junction, PCC refers to the posterior cingulate cortex, and VS refers to the ventral striatum.

An important mechanism potentially underlying information transmission involves the contributions of the brain network and the social network. The multilayer brain-social network integrates these two networks that capture the cognitive and behavioral processes during information transmission. The aim of the multilayer brain-social network proposed by Falk and Bassett (2017) is (1) to understand the reason why some people have a greater tendency to share ideas, (2) to explain how individuals are affected and assimilated by others’ opinions and behaviors and (3) to clarify how information is transmitted from one person to another through the social network. This network has two layers (see Figure 2). The first is the neural network layer. Multiple brain regions collaboratively participate in the processing of information from other regions and decide whether to transmit it to others. The neural networks mainly consist of the default network responsible for cognition and information processing (e.g., mPFC), the mentalizing network that processes the intentions of others (e.g., the TPJ and PCC), and the reward system responsible for judging whether information transmission will produce a reward [e.g., the ventral striatum (VS) and vmPFC; Falk and Bassett, 2017; Scholz et al., 2017; Falk and Scholz, 2018]. The second layer is social networks, which are composed of interconnections among individuals and are responsible for spreading information.

In multilayer brain-social networks, information is first processed at the neural network layer of an individual to form a solid idea or behavior and then transmitted to others through social networks to synchronously activate others’ neural networks and change their ideas or behaviors. This process is essentially a brain-person connection (Falk and Bassett, 2017). In addition, information transmission in multilayer brain-social networks is affected mainly by two factors. First, an individual’s neural networks determine whether to spread ideas or behaviors to others. The reward-related VS and vmPFC are the key structures for spreading ideas or behaviors. Individuals who like to share information have higher activation levels in these two brain regions when they decide to share information with others, suggesting that the VS and vmPFC determine the perceived reward value of sharing information and then decide whether an idea or behavior should be spread in a social network (Baek et al., 2017; Scholz et al., 2017). Second, neural networks and social networks determine the information that is transmitted and to whom in a social network. Individuals with similar brain processing patterns are more inclined to share ideas or behaviors with each other (Mobbs et al., 2009; Fareri and Delgado, 2014; Falk and Bassett, 2017). Parkinson et al. (2017) found that two individuals with a close relationship in a social network had highly similar whole-brain activity when watching the same movie clips and that the similarity decreased with the increase in social distance between the two. Furthermore, the similarity in whole-brain activity between two individuals predicted their social distance and determined whether they could become friends and share ideas and behaviors (Parkinson et al., 2017). In addition, social status can influence the flows of information in which individuals with a high status have a greater capacity to decide whether to deliver information to other people and low-status individuals are more likely to be impacted by high-status individuals’ attitudes, emotions or behaviors (Hussong et al., 2019). This observation suggests that neural networks may influence information processing and an individual’s tendency to share, whereas social network construction may influence the direction of spreading information.

Previous studies only focused on the neural or social network, and rarely associated the social network with the neural network. Unfortunately, neither social networks nor neural networks provide sufficient descriptions of the dynamic transmission of information. Multilayer brain-social networks integrate the neural networks and social networks of individuals and more comprehensively illustrate how information transmission is influenced by the brain and social environment. On the one hand, neural networks influence individuals’ tendency to spread their ideas or behaviors in a social network by influencing the reward value of sharing information. On the other hand, social network construction also affects information dissemination. Thus, multilayer brain-social networks not only are related to synchronous brain activity among people in the network but also emphasize that the essence of information transmission is actually the interaction between the brain and the social environment. However, the exploration of multilayer brain-social networks is still in an early stage, and the causal relationship and the potential influencing variables involved must be further clarified.

## Neurochemical Bases of the Social Network

### Oxytocin, β-Endorphin, Dopamine, and Social Network Structure

One of the most widely studied topics with regard to social networks and the brain is the endocrine system and genetic profile. Two recent studies by Pearce’s group directly investigated the relationship between social network structure and the endocrine system and genes, which are the most systematic and detailed studies performed to date (Pearce et al., 2017, 2018). In these two studies, the researchers genotyped 33 single nucleotide polymorphisms (SNPs) from seven genes, including *OXTR* (oxytocin receptor gene), *AVPR1A* (vasopressin receptor gene), *OPRM1* (mu opioid receptor 1, a β-endorphin receptor gene), *DRD1* and *DRD2* (dopamine receptor genes), *ANNK1* (dopamine receptor gene), and *HTR1A* and *HTR2A* (serotonin receptor genes). The links between these genes and social relationships (such as empathy, romantic relationships and sociality) have been confirmed by many studies, but most of these studies focused on dyadic relationships (Rodrigues et al., 2009; Troisi et al., 2011; Uzefovsky et al., 2015; Nummenmaa et al., 2016). Significant relationships mainly focus on *OXTR*, *DRD2*, *OPRM1* and the social network size.

The neuropeptide OXT (oxytocin) plays an important role in the social life of humans (Liu et al., 2019). OXT-related genes, including SNPs in the *OXTR* gene, widely act on multiple brain regions (e.g., the amygdala and insula) to affect an individual’s social interaction, empathy, and emotion regulation, which are closely related to social network construction (Pearce et al., 2018). For instance, individuals with the TT/TG genotype of rs1042778 in the *OXTR* gene have larger social networks than those with GG the genotype at that locus. Through structural equation modeling, researchers further found that the rs1042778 polymorphic variation increased the network size by reducing an individual’s negative affect and social inhibition (Creswell et al., 2015). Similarly, some recent studies also found that the genotype of rs2268498 in the *OXTR* gene was associated with the social network size. Individuals with the TT genotype of rs2268498 had a larger social network than those with the CC/CT genotype at that locus (Sariyska et al., 2018). However, Pearce et al. (2017) examined the association between 11 *OXTR* SNPs and social network size and identified a small number of *OXTR* SNPs that were linked to wider social network size. Specifically, only the genotype of rs237887 in the *OXTR* gene was confirmed in studies by Pearce et al. (2017, 2018) in the general population. Researchers also found that the effects of variation in OXTR SNPs disappeared after controlling for endorphins, suggesting that the emphasis on oxytocin in many dyadic social relationships may not be as important in social network construction compared to endorphins (Pearce et al., 2017).

In addition to OXT, researchers increasing focused on the role of β-endorphin, a member of the endogenous opioid peptide system, in the agreeableness and social connection of mammals (Johnson and Dunbar, 2016). In general, β-endorphin not only relieves both physical and social pain but also affects the social network structure of individuals by regulating social motivation and the perceived positive value of social connections (Johnson and Dunbar, 2016). Johnson found that pain tolerance was positively correlated with the social network size, indirectly indicating that individuals with a higher activation level of the opioid peptide system often have more social connections (Johnson and Dunbar, 2016). Pearce and colleagues found that the *OPRM1* rs1799971 variant in β-endorphin receptors was closely related to an individual’s ability to blend into social networks. Individuals with the AA genotype of rs1799971 in the *OPRM1* gene usually blended themselves better into larger social groups than those with a different genotype at that locus. One explanation is that the *OPRM1* variant affects an individual’s assessment of social reward value, thereby affecting individual social preferences (Pearce et al., 2017, 2018).

Dopamine and polymorphisms in the dopamine receptor 2 gene (*DRD2*) also affect the social network structure of humans. In a positron emission tomography (PET) imaging study, *DRD2* gene polymorphisms were shown to regulate the synthesis and release of dopamine, and dopamine further regulates the intensity of empathy responses and agreeable behavior and the degree of attachment by acting on multiple brain regions, including the amygdala, vmPFC, and ACC (Atzil et al., 2017). The link between *DRD2* gene polymorphisms and social network construction and maintenance was further confirmed in other studies. The genotype of rs4648317 in the *DRD2* gene is significantly correlated with the social network size and how well the individual blends into social groups (Pearce et al., 2017, 2018).

The research on genes and social networks is still in the preliminary stage, and the verification and integration of different receptor genes on the prediction of social network structure must be further strengthened. In addition, the current social network research is less able to combine studies of genes with the brain. Two studies on group-housed rats found that individual variation in dominance status and social network position were associated with gene expression in the brain. For instance, So et al. (2015) revealed that more dominant individuals have higher levels of corticotropin-releasing factor (*CRF*) mRNA in the amygdala and hypothalamus, and higher levels of glucocorticoid receptor (*GR*) and brain-derived neurotrophic factor (*BDNF*) mRNAs in the hippocampus, whereas social network position did not show any relationship with gene expression in the brain. The studies discussed above confirmed the close relationship between the amygdala and hippocampus with social status. The expression of CRF, GR, and BNDF in these regions has been shown to promote learning and evaluation of social dominance information. Williamson et al. (2016) reported that social network position (e.g., out-degree and out-closeness, which is the total paths from an individual out to all of the other individuals in the network) was negatively associated with levels of the *DNMT1* and *DNMT3a* mRNAs in the hippocampus, which are two neural plasticity genes linked to social competence, learning and memory. However, consistent results for the social network and gene expression have not been obtained. Further studies are necessary to verify and expand the related results.

However, numerous studies on the neurobiological basis of social status focus on very different aspects. Low social status is thought to increase the physiological effects of chronic social stress (Simons and Tung, 2019). Researchers highlighted various aspects of serotonin related to social stress (Willard and Shively, 2016). Accordingly, researchers have found that the social status of female non-human primates is negatively correlated with the levels of the serotonin-1A (5-HT1AR) receptor and serotonin transporter (5-HTT), both of which regulate the synaptic effects of serotonin (Asher et al., 2013; Embree et al., 2013; also see review in Willard and Shively, 2016). Low social status has also been shown to be associated with enhanced hypothalamic-pituitary-adrenal (HPA) axis function, which is important for social stress sensitivity. For example, in a study using naked mole rats, the density of the CRF receptor involved in HPA axis regulation, particularly in the piriform cortex and cortical amygdala, was higher in subordinates than in superiors (Beer et al., 2016). In another study in rhesus macaques, low-status animals had increased levels of glucocorticoids (GCs) and related gene expression, which is thought to be regulated by social stress-induced HPA axis activities (Snyder-Mackler et al., 2019). Human studies reported inconsistent results. For instance, Murray et al. (2019) revealed that lower subjective social status was not associated with the differential expression of any genes involved in HPA axis-related GR signaling. Other studies examined the effects of *OXTR*, vasopressin 1a receptor and *DRD2* genotypes on social status (Shanahan et al., 2008; Lee et al., 2019). However, few studies have compared the similarities and differences in gene expression between individuals with different social statuses and social network positions. Social network studies have emphasized the benefits of social relationships, whereas social status studies emphasized social stress. Future studies are needed to integrate these two research directions and build a more comprehensive and deeper understanding of social context.

### OXT and Social Network Function

Information transmission in social networks also depends on the neuroendocrine system, especially the neuropeptide OXT. OXT plays an extremely important role in social group life by regulating information transmission and emotional contagion among people. Spengler et al. (2017) observed increased OXT secretion in both the imitator and the imitated when individuals made the same facial expression as their friend. Compared with the control group, the individuals who were intranasally administered OXT usually experienced an increased perceived intensity of their friend’s facial expression, suggesting that the increased OXT level enhanced emotional transmission (Spengler et al., 2017). In addition, *OXTR* gene polymorphisms affect an individual’s facial expression recognition ability (e.g., rs237887 and rs2268490; Kim H. W. et al., 2019), theory of mind (e.g., rs53576; Wu and Su, 2015), and prosocial ability (e.g., rs13316193, rs1042778, and rs237887; Wu and Su, 2018), among other processes, which are closely related to an individual’s ability to transmit and receive social information. However, more direct studies are needed to explore the relationship between genes and the functional attributes of social networks.

## Discussion

Establishing social connections and participating in social networks are crucial to human survival (Fareri and Delgado, 2014). Evidence mapping multiple layers of neurophysiological mechanisms underlying social networks has highlighted the fact that social network structure and function are co-regulated by a gene-endocrine-brain circuit. Although the studies mentioned above reported various findings, these results and theoretical implications might be integrated.

The construction and maintenance of social networks depend on the interaction of multiple neural systems. In the context of previous neuroimaging and neurobiology studies in humans and animals, the amygdala functions as a hub for the social brain that supports the coding of emotion-related social signals and assessment of social reward value, and it is central to handling the needs of complex social life (Bickart et al., 2014). In addition, based on the evidence discussed above, the necessary social network construction presumably relies on social cognition and its two main mechanisms: the mentalizing network is involved in inferring the mental state of others, and the mirror network is assumed to observe, self-perform, imitate and imagine biological actions (Oberman et al., 2007). However, a plausible hypothesis is that these neural systems’ support of the social network is correlated. On the one hand, according to resting-state fMRI studies, social network structure is positively predicted by the functional connectivity between the amygdala and the mentalizing network and the functional connectivity among brain regions of the mentalizing network. On the other hand, in terms of social network function, interpersonal information transmission depends on the correlations among neural networks, especially the default network, reward system and mentalizing network. Constructing and maintaining a social network position requires individuals to learn about multiple social relationships with other individuals and to be able to express socially and contextually appropriate behavior to all other individuals within their social network (Williamson et al., 2016). Therefore, individuals with complex social networks (e.g., a large social network size, high social network density, high in-degree or high out-degree) likely have more power and social resources. They require not only greater social cognitive abilities to help them process various social signals but also greater general cognitive ability to memorize and differentiate the relationships, names and faces to deal with their complicated social context. Therefore, the individuals at the core of the social network usually exhibit higher neural activity in the related brain regions. Taken together, these findings provide a powerful framework for the social network structure and function that depends on the functions of complex neural systems.

In addition, current research focuses on the neurobiological basis of social networks. Neuropeptides, particularly OXT, have been viewed as important for identifying emotions, pair bonding and parental care during social interaction (Kanat et al., 2014). OXT is a promising target for social interaction because the amygdala is one of the core nodes of OXT action in the brain (Bickart et al., 2014; Pearce et al., 2018). For example, the volume of the amygdala mediates the effect of the *OXTR* rs53576 genotype on an individual’s level of trust in others (Nishina et al., 2018). In a review, *OXTR* SNPs were suggested to affect social cognition by modulating the anatomy and function of the social brain network, e.g., amygdala, dACC and hypothalamus (Meyer-Lindenberg and Tost, 2012; Kumsta and Heinrichs, 2013; Kanat et al., 2014). A plausible hypothesis is that OXT may modulate the relationship between brain activities and social network function. Future research could directly inject OXT or change the expression of the *OXTR* gene in the social brain network using electrophysiological technology in animal studies to verify whether OXT affects social networks (including the quantity and quality of social relations, social status, information transmission, etc.) and the function of the social brain network. However, some genes closely related to the social network are not associated with brain regions related to the social network, such as *DNMT1* and *DNMT3a*. The structure of the social network in rats is related to the mRNA expression of *DNMT1* and *DNMT3a* in the hippocampus (Pearce et al., 2017), whereas the relationship between the hippocampus and the social network has not yet been confirmed in previous studies. On the one hand, the hippocampus is closely related to memory. However, few researchers have examined the role of hippocampus in the construction and maintenance of social networks. On the other hand, the activities of the brain and endocrine system may not be synchronous. Due to the plasticity of the brain, the endocrine system may change the function of the brain under long-term stimulation from the social environment. Thus, the endocrine system may affect the social network structure by shaping the structure and function of the “social brain” (Rebuli et al., 2017). However, social network-related gene research has only recently attracted the attention of researchers and is still in its infancy. Many findings must be reported and confirmed. At this time, the correlation between the endocrine system and the function of brain regions should be highlighted. Future studies should expand relevant research to explore how the endocrine system, genes and the brain interact. Moreover, most of the studies focus on only one neuropeptide or simply list the functions of several neuropeptides but few focuses on how the interaction of different neuroendocrine systems affects social networks. Because different endocrine systems (e.g., oxytocin, dopamine, endorphin, etc.) are regulated by social behavior, they may play distinct roles in social behaviors, according to previous studies. For instance, OXT is important for pair formation (e.g., romantic relationship and parent-child relationship), since OXT stimulates social contact in both human and animals (Witt et al., 1992; Kosfeld et al., 2005; see Dunbar, 2010 for a review). The maintenance of stable and long-term social connections may depend on the endorphin system, which is vital to experiencing interpersonal warmth and attachment (Machin and Dunbar, 2011; Pearce et al., 2017). Therefore, the effect of the interaction of different endocrine systems on the construction and maintenance of social networks may be reflected in both function and time axes. However, more direct studies are needed to explore this hypothesis in the future.

The neurophysiological mechanisms involved in social status were discussed. Although social status and social network structure are strongly correlated according to previous studies, they represent different attributes of social structure. Social status mainly focuses on the influence of social power and economic level on the capacity of social resource control, while the social network focuses more on the emotional support and information support provided by social relations. According to the literature discussed above, the social status and social network also differ in the brain mechanism, neuroendocrine system, and gene expression. Social status and the social network are very different research fields. Previous studies emphasized the effect of social status on the brain activities related to cognitive function, while social network research mainly focused on brain networks related to social cognition (Kanai et al., 2012; Farah, 2017). Based on neurobiology, social status is usually linked to stress-related endocrine and receptor genes, yet studies on social networks still focus on the role of social relationship-related endocrine and receptor genes in network construction and maintenance (Willard and Shively, 2016; Pearce et al., 2017). In the future, more studies should compare the neurobiological bases of the social network and social status or explore the interaction between them to better understand the different aspects of individual social structures.

In summary, social network structure and function are regulated by genes, the endocrine system and the brain. Existing studies are mostly based on a single factor, i.e., genes, the endocrine system, or brain; hence, future research should combine these factors to construct a more comprehensive theoretical neural model of social networks.

## Future Directions and Conclusion

Although significant progress has been achieved in determining the neural mechanisms of social networks, many questions remain to be addressed. Most existing studies have focused on understanding the structure of social networks from the perspective of the social cognitive ability of individuals and have paid little attention to the effect of memory abilities on social network structure. In contrast to pure social interaction, social networks are more complex and may involve more cognitive and processing activities. A social network is an aggregate of various types of social interaction behaviors. Building and maintaining a large-scale and complex social network requires an individual to have not only strong social functions but also a strong memory ability to store a very large amount of social network information. On the one hand, memorizing high-quality social relationships and filtering irrelevant social relationships will help individuals quickly identify people who might be beneficial or harmful to them. On the other hand, if an individual is unable to extract a social relationship from memory, the whole social network might be perceived differently, potentially leading to wrong judgments and decisions (Brashears and Quintane, 2015; Meyer et al., 2015). Therefore, future studies should focus on whether memory abilities limit social network structure and how social network structure is stored in the brain. In a related study, the memory performance of participants in social information-related memory tests was positively correlated with the size of their social support networks, and strong activation of the dmPFC and mPFC during memory testing was also positively related to the network size of the participants (Krol et al., 2018). However, the study simply measured the individuals’ ability to store the trait information of friends, without exploring the storage patterns of network structures. For example, individuals with a complex network structure may simplify the network structure by storing social network information using ternary relationships as a unit, whereas individuals with a simple network structure tend to use binary relationships as a unit for storing social network information; moreover, the brain activities involved in the construction of new social networks may vary depending upon individuals’ different social network structures. The key to exploring the reason why an individual prefers a specific social network structure is to first understand how the individual thinks about and processes social networks. Therefore, the theoretical neural model of social networks must be assessed from the perspective of memory ability and, more extensively, the cognitive level.

Moreover, additional studies should be conducted to explore the theoretical neural model of social networks in patients with mental illnesses. Using behavior indicators, a large number of studies have confirmed that patients with a mental illness generally construct network structures that are different from those constructed by people without a mental illness. For example, in a study investigating networks in 10 areas stricken by fire, researchers found that the individuals with posttraumatic stress disorder (PTSD) symptoms generally had a low in-degree ties and that their friends did not have social ties with each other (Rosenquist et al., 2011; Bryant et al., 2016). In contrast, individuals with depression often had lower out-degree ties, and depressive symptoms were spread among members in their social networks (Rosenquist et al., 2011; Bryant et al., 2016). Based on these results, patients with different mental illnesses may present different network structure abnormalities. Mental illness can also change brain function and endocrine function. For example, PTSD causes disorders of the HPA axis, such as decreased cortisol levels, downregulated type I glucocorticoid receptor mRNA expression, and upregulated type II glucocorticoid receptor mRNA expression (Yamamoto et al., 2010); severe depression causes dysfunction of the limbic system-cortex-striatum-globus pallidus-thalamic circuit (Bora et al., 2012). However, researchers have not clearly determined whether changes in neural mechanisms and endocrine function caused by mental illness are affected by the social network structure. From the perspective of social network function, the changes in brain function and the endocrine system caused by mental illness alter the tendency of an individual to spread or accept others’ ideas and behaviors by affecting the individual’s social anxiety, emotional state, and perceived social support, among other factors (Falk and Bassett, 2017). If sufficient evidence at the behavior and neural mechanism levels is obtained to show that the social networks of an individual affect the occurrence, development and recovery of mental illnesses, then social network indicators may serve as reference indicators for diagnosing certain mental illnesses. These indicators would be very important in the prevention and treatment of mental illness.

In addition, social networks are dynamic. Studies should be conducted to further explore the synergistic changes between social networks and neural mechanisms, which might provide causal or dynamic evidence for the theoretical neural model of social networks (Sallet et al., 2011; Hatch et al., 2013). Although existing studies have examined the long-term disappearance and reconstruction of social network connections with time and life events, few studies have explored the relationship between the development of social networks and changes in brain function. A study conducted by Sallet et al. (2011) on primates was the first to explore the causal relationship between social networks and brain structure from the perspective of causality. In the study, 23 monkeys were housed in different environments with different population sizes to simulate human individuals with different social network sizes. Monkeys with larger social network sizes had larger gray matter volumes in the STS and PFC and stronger functional connections between the frontal and temporal lobes (Sallet et al., 2011). Although the study was not longitudinal, it clearly showed that social networks change the function and structure of the brain, providing preliminary evidence for future research. Future studies could address the three research directions described below. First, using data related to social networks and the neural system of individuals of different ages, the question of whether network development depends on the maturity of the brain can be clarified. Second, by collecting social network data for individuals in stages when they enter a new environment (e.g., when a student goes to college), changes in brain function during the establishment and development of new networks can be described. Third, a dynamic social network may result from long-term changes in gene expression mediated by epigenetic modification. For example, either negative/adverse experiences or a favorable environment has recently been shown to generate neurobiological changes (e.g., DNA methylation). These changes further affect the receptor expression that alter the sensitivity to hormones guiding social behavior (Shepard et al., 2009; Jeyaraj et al., 2020). Thus, an interesting approach would be to consider how life experience changes long-term individual social networks from an epigenetic perspective. Fourth, using animals as study subjects, the causal relationship between social networks and the brain can be investigated. On the one hand, brain activities and endocrine systems can be easily manipulated in animals using neuroelectrophysiological approaches. For example, researchers can use optogenetics to regulate the activity of neurons and explore the effect of brain activities on social structure by regulating the excitability of neural circuits in specific brain regions; on the other hand, animal research is conducive to long-term tracking which can help researchers distinguish the cause and effect of interactions among social environment, endocrine, and genes. Additionally, animal studies of rodents and non-human primates using automated tracking technologies revealed that these animals live in large groups with hierarchically organized social structures (So et al., 2015; Williamson et al., 2016). Thus, studies exploring the neurobiological mechanism of the social network in laboratory animals are feasible.

In conclusion, the combination of social networks and neuroscience is a research hotspot in social cognitive neuroscience. Currently, a number of studies have identified the neurobiological indicators that correspond to behavioral indicators of social networks. Future research should explore the neurobiological mechanisms of social networks and the significance of social networks in clinical practice; studies addressing these topics will improve our understanding of the effect of social factors on the mental health of individuals and provide researchers with new insights into the clinical diagnoses and treatment of individuals with mental illnesses.

## Author Contributions

All the authors contributed substantially to the manuscript and approved the content of the manuscript. YS contributed to conceiving and designing the writing framework and revised the manuscript. MH reviewed the literature and drafted the manuscript. GJ and HL reviewed the literature and revised the manuscript.

## Conflict of Interest

The authors declare that the research was conducted in the absence of any commercial or financial relationships that could be construed as a potential conflict of interest.
